# Molecular ion battery: a rechargeable system without using any elemental ions as a charge carrier

**DOI:** 10.1038/srep10962

**Published:** 2015-06-04

**Authors:** Masaru Yao, Hikaru Sano, Hisanori Ando, Tetsu Kiyobayashi

**Affiliations:** 1 Research Institute of Electrochemical Energy, Department of Energy and Environment, National Institute of Advanced Industrial Science and Technology (AIST) 1-8-31 Midorigaoka, Ikeda, Osaka 563-8577, Japan

## Abstract

Is it possible to exceed the lithium redox potential in electrochemical systems? It seems impossible to exceed the lithium potential because the redox potential of the elemental lithium is the lowest among *all* the elements, which contributes to the high voltage characteristics of the widely used lithium ion battery. However, it should be *possible* when we use a molecule-based ion which is not reduced even at the lithium potential in principle. Here we propose a new model system using a molecular electrolyte salt with polymer-based active materials in order to verify whether a molecular ion species serves as a charge carrier. Although the potential of the negative-electrode is not yet lower than that of lithium at present, this study reveals that a molecular ion can work as a charge carrier in a battery and the system is certainly a molecular ion-based “rocking chair” type battery.

Lithium-based rechargeable batteries, *i.e.*, lithium-ion batteries, have been widely used as a power source for electronic devices because these battery voltages are generally high compared to other batteries. This characteristic stems from the fact that the redox potential of elemental lithium is the lowest among *all* the elements. In other words, it seems in principle impossible to go beyond the lithium potential. However, we consider that it should be *possible* when we abandon the preconceived notion that the charge carriers must be elemental ions such as Li^+^. Namely, if we can find a molecule-based ion which is not reduced even at the lithium potential, we will be able to overcome the limit of the lithium potential and develop a very high voltage battery. To realize such a molecular ion battery, the first requirement is to demonstrate that a rechargeable system is viable in which a single molecular ion species serves as a charge carrier.

To build such a molecular ion battery system, the search for electrode active materials which can electrochemically store molecular ions is inevitable. One of the candidate categories is a series of redox active organic materials, since they tend to be highly adaptable to ions^1^,^2^,^3^,^4^,^5^,^6^,^7^,^8^,^9^. In fact, we have reported various organic active materials for use in lithium-based rechargeable batteries^5^,^6^,^10^,^11^,^12^, and discovered that some of them can work even in sodium and magnesium electrolyte systems^7^,^8^,^9^. In addition, certain types of polymeric compounds are known to insert and deinsert certain molecular anions, not Li^+^, accompanied by their redox reaction^13^,^14^,^15^,^16^. Polyaniline, polypyrrole and polythiophene, known as classical π-conjugated conductive polymers, are the representatives, and have been studied since the 1980’s^13^,^14^. This kind of redox properties have been applied to a positive electrode reaction in rechargeable lithium batteries^14^,^17^,^18^,^19^,^20^,^21^. In this type of battery, the Li^+^ insertion/deinsertion takes place at the negative electrode. However, so long as we rely on the Li^+^-based reaction at the negative electrode, a severe concentration change in the electrolyte salt during the charge/discharge process occurs, which induces a problem in realizing a practical battery using these active materials. Therefore, it is indispensable to develop the so-called “rocking chair” type system for the charge carrier, in which the identical charge carrier is exchanged between the positive and negative electrodes. This paper describes a concept and preliminary performance of our molecular ion battery system and its further prospects.

## Results

### Design of electrode active materials

**Figure 1:**
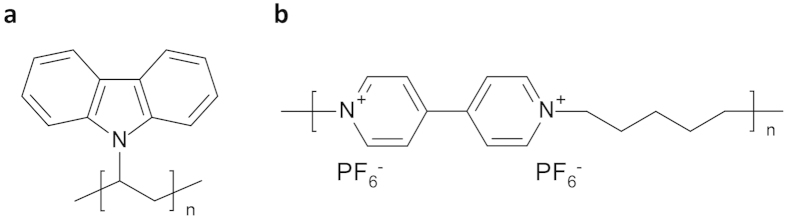
Chemical structures of the organic active materials. (**a**), Positive electrode, poly(*N*-vinylcarbazole) (PVK). (**b**), Negative electrode, poly(1,1’-pentyl-4,4’-bipyridinium dihexafluorophosphate) (PBPy).

To construct a “rocking chair” type molecular ion battery, we used redox polymers. As the positive electrode material, a redox polymer having the carbazole skeleton, poly(*N*-vinylcarbazole): PVK (Fig. 1a)^19^,^20^, which we previously reported as the positive electrode material in a lithium electrolyte system^21^ was used. As for the negative electrode active material, we focused on a 4,4’-bipyridinium moiety, known as the viologen unit, and synthesized a polymer bearing the bipyridinium units by polymerization through the quaternization reaction^22^,^23^ of the nitrogen atoms of 4,4’-bipyridine with dibromopentane, followed by the ion exchange from Br^−^ to PF_6_^−^ to yield poly(1,1’-pentyl-4,4’-bipyridinium dihexafluorophosphate): PBPy (Fig. 1b). The ^1^H-NMR analysis indicated that the average degree of polymerization is 30 to 50.

### Charge/discharge performance of each electrode active material

**Figure 2:**
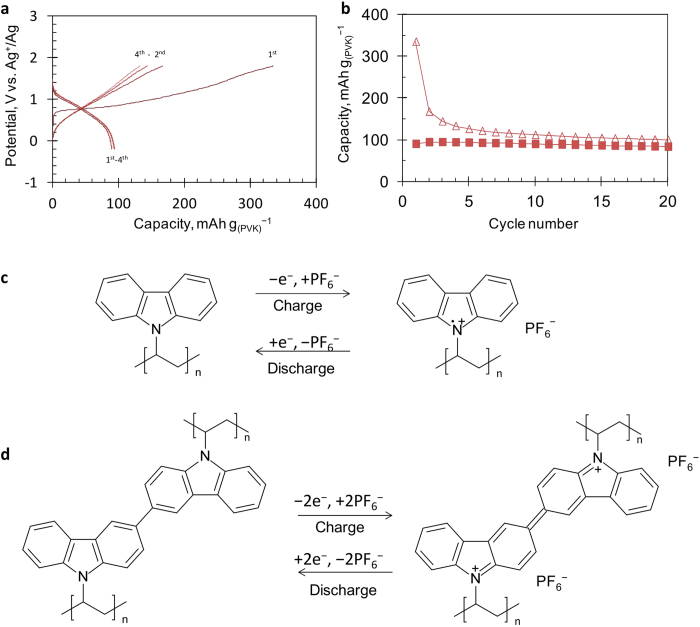
Charge/discharge performances of the electrode using PVK in a three-electrode cell. (**a**), Charge/discharge curves of the PVK-electrode. (**b**), Cycle-life performance of the PVK-electrode (Δ: charge, ■: discharge). Conditions, electrolyte solution: 1 mol L^−1^ *n*-Bu_4_NPF_6_/PC, current density: 100 mA g^−1^, potential range: −0.2-+1.8 V *vs.* Ag^+^/Ag. (**c**), Possible charge/discharge reaction of the PVK. (**d**), Possible charge/discharge reaction of cross-linked PVK.

Figure 2a shows the first several charge/discharge, *i.e.,* the PF_6_^−^ insertion and deinsertion, respectively, curves of the prepared PVK electrode in the *n*-Bu_4_NPF_6_ electrolyte system in a three-electrode setup with the Ag^+^/Ag reference electrode. The electrode exhibits smooth discharge curves with an intermediate potential of 0.8 V *vs.* Ag^+^/Ag with the discharge capacity of about 90 mAh g_(PVK)_^−1^. The obtained capacity is 65% of the theoretical value of 139 mAh g_(PVK)_^−1^ based on the assumption that a one-electron transfer redox reaction occurs between the neutral state and cation radical state of the carbazole moiety as shown in the reaction equation of Fig. 2c. The electrode using PVK shows no appreciable decay in the discharge capacity upon cycling (Fig. 2b) and maintains 84 mAh g_(PVK)_^−1^ after 20 cycles. The coulomb efficiency (discharge capacity/charge capacity) during the early cycling is poor; however, it gradually becomes better upon cycling, reflecting the coupling reaction between the carbazole moieties during the charge process as explained in some previous reports^19^,^20^,^21^. The cross-coupled PVK shown in Fig. 2d can also contribute to the charge/discharge process in the electrode. As reported by some groups, the 3- and 6-positions of the oxidized carbazole moiety have a relatively high spin density and these sites are reactive^19^,^20^,^21^,^24^,^25^,^26^. Chemical modifications of such reactive positions by some protecting groups will stabilize the oxidized state, which may improve the coulomb efficiency during the charge/discharge process.

**Figure 3:**
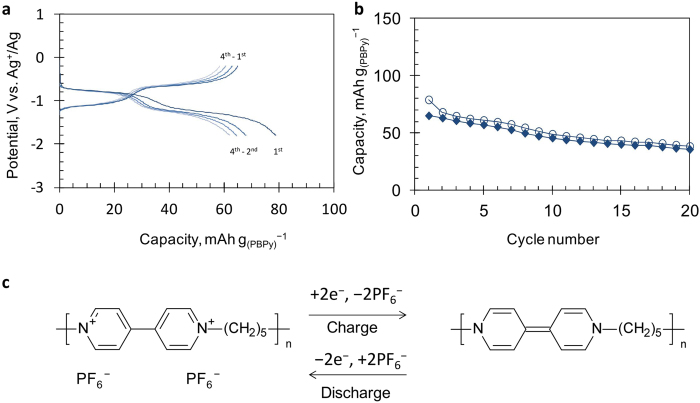
Charge/discharge performances and possible reactions of the electrode using PBPy in a three-electrode cell. (**a**), Charge/discharge curves of the PBPy-electrode. (**b**), Cycle-life performance of the PBPy-electrode (○: charge, ♦: discharge). Conditions, electrolyte solution: 1 mol L^−1^ *n*-Bu_4_NPF_6_/PC, current density: 100 mA g^−1^, potential range: −1.8-−0.2 V *vs.* Ag^+^/Ag. (**c**), Possible charge/discharge reaction of PBPy.

The charge/discharge, *i.e*., the PF_6_^−^ deinsertion/insertion, curves of the negative electrode using the synthesized polymer, PBPy, are shown in Fig. 3a. These curves consist of two plateau potential regions at around −0.7 and −1.2 V *vs*. Ag^+^/Ag, which are considered to reflect the stepwise two-electron transfer redox reaction of the bipyridinium moiety. The capacity of 79 mAh g_(PBPy)_^−1^ observed in the first charge is 76% of the theoretical value of 104 mAh g_(PBPy)_^−1^ based on the assumption of the two-electron redox reaction of the repeating unit (Fig. 3c). (In this paper, the charge and discharge of the negative electrode refer to the reduction and oxidation processes of the polymer, respectively). Although the capacities of the electrode for both the charge and discharge processes decrease upon cycling and the coulomb efficiency is even low as shown in Fig. 3b, the electrode maintains 36–38 mAh g_(PBPy)_^−1^ after 20 cycles. The degree of polymerization for the PBPy polymer prepared in this study is not high as described above; therefore, the solubility of the PBPy polymer in the ordinary organic solvents is still relatively high. In fact, the electrolyte solution after cycling was colored and a migration of some organic compounds was also observed, indicating a dissolution of organic active materials occurs during cycling. Such a phenomenon can be a reason for the capacity decay of the electrode as reported for many low-molecular-weight organic active materials^3^,^10^,^11^,^12^. In addition to the chemical modifications of the bipyridinium moiety which can increase the chemical stability of the skeleton itself, controlling some polymeric structures such as branching and molecular-weight-increase may improve the cycle stability of the PBPy-electrode.

### Elucidation of the carrier ion

**Figure 4:**
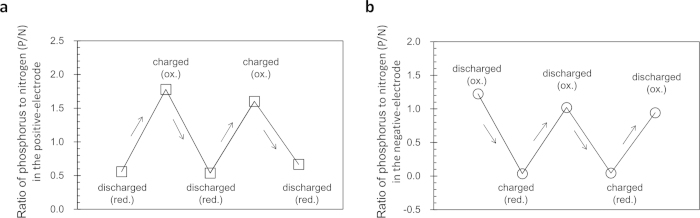
Change in stoichiometric ratio of phosphorous to nitrogen in the electrodes during cycling. (**a**), PVK-based positive electrode. (**b**), PBPy-based negative electrode. The y-axis value, *i.e.* P/N ratio was calculated using the relative signal intensity of the phosphorous atoms to the internal reference peaks obtained from the EDX measurement. In this figure, the charged and discharged states of the positive electrode refer to the oxidized (ox.) and reduced (red.) states for PVK, respectively. The negative electrode is the other way around, *i.e*., the charged and discharged states of the negative electrode correspond to the red. and ox. states for PBPy, respectively. For details, see the experimental part and Supplementary Figures S1 and S2.

In order to prove the concept of the molecular ion battery, it is important to show that the PF_6_^–^ ion is indeed inserted in and extracted from these active materials during the charge/discharge process; therefore, we evaluated the change in stoichiometry of PF_6_^−^ in the electrode ex situ by energy dispersive X-ray (EDX) spectroscopy. Figure 4 plots the stoichiometric ratio of the phosphorus to nitrogen (P/N), *i.e.*, the ratio of PF_6_^−^ per carbazole unit in the positive electrode or that per pyridine unit in the negative electrode during the charge/discharge process. (The charge/discharge curves of the prepared cells and the EDX spectral changes are shown in Supplementary Figures S1 and S2, respectively.) As for the positive electrode (Fig. 4a), the P/N ratio increases after the charge process, and the ratio decreases when the electrode is discharged. As for the negative electrode (Fig. 4b), the PF_6_^−^ concentration decreases after the charge process, and returns to the initial level when the electrode is recharged, which is a complementary behavior to that of the positive electrode. The observed ratio changes in both electrodes are considered to reflect the charge/discharge mechanisms of these active materials suggested in Figs. 2c,d and 3c. The stoichiometric changes are slightly overestimated; however, they are in good agreement in the order of magnitude, providing evidence that the charge carrier in this system is PF_6_^−^ and the prepared system becomes certainly a PF_6_^−^-based “rocking chair type” battery.

### Battery performance of the full cell

**Figure 5:**
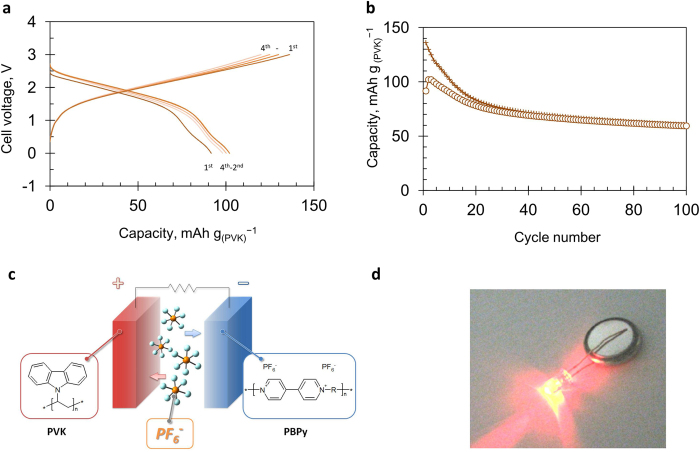
Performance of the molecular ion battery composed of PVK, PBPy and *n*-Bu_4_NPF_6_. (**a**), Charge/discharge curves of the prepared full cell. Electrolyte solution: 1 mol L^−1^ *n*-Bu_4_NPF_6_/PC, Current density: 100 mA g_(PVK)_^−1^, Voltage range: 0.0-3.0 V. (**b**), Cycle-life performance of the cell (+: charge, ○: discharge). (**c**), Conceptual drawing of the mechanism of the prepared molecular ion battery. (**d**), LED lamp powered by the prepared molecular ion battery.

Finally, the battery performance of the full cell consisting of the PVK positive electrode and the PBPy negative electrode is described. In assembling the cell, the capacity of the negative electrode was adjusted to be more than twice that of the positive electrode in order to utilize the lower potential region of the negative electrode. Figure 5a shows typical charge/discharge curves of the prepared sealed full cell. The cell exhibited about 100 mAh g_(PVK)_^−1^ with the intermediate potential difference of 1.8 V. The obtained discharge capacity is close to the value found in the three-electrode test of the PVK-electrode. The observed voltage well agrees with the difference between the potential of the discharge curve for the PVK-electrode (0.8 V *vs*. Ag^+^/Ag) and the lower potential of the discharge curve for the PBPy electrode (−1.1 V *vs*. Ag^+^/Ag). The observed potential difference is also in agreement with the theoretically estimated energy level difference (1.98 eV) obtained from a quantum chemistry calculation based on the density functional theory (DFT), in which simplified model compounds were used (Supplementary Figure S3). This calculation also supports the fact that the reaction mechanism of this system is based on the redox of the present organic active materials. As for the prepared molecular ion battery system, the conceptual drawing of the mechanism and a demonstration to drive an LED lamp are shown in Fig. 5c,d. (The results of a high rate test of the cell are described in Supplementary Figure S4.) The prepared full cell exhibited a fairly good cycle stability; the cell maintained about 65% of the initial capacity after 100 cycles as shown in Fig. 5b.

## Discussion

The present study has demonstrated the feasibility of a rechargeable molecular ion battery based on a model system with PF_6_^–^ as the charge carrier, and PVK and PBPy as the positive and negative electrodes, respectively. When successful in further developing the molecular ion battery, the benefits we would gain are:

### Breaking through the Li Potential Limitation

As explained in the introduction of this paper, when we find an active electrode material of which the redox potential is lower than that of lithium and if a molecular charge carrier tolerates that low a potential, we can break through the lower limit of –3.0 V *vs.* SHE. Thereby we may be able to develop a higher voltage battery than the current lithium ion batteries.

### High Ion Conductivity in the Electrolyte Solution

Molecular ions tend to show a high limiting molar conductivity, *e.g.* PF_6_^−^ has more than twice the limiting molar conductivity of Li^+^ in ordinary organic solvents, since these molecular ions do not suffer from any solvation effect^27^,^28^. A battery, which can be charged or discharged in a very short time, may become available using these characteristics.

### Free from the Dendrite Risk

In the current lithium battery systems, the dendrite formation, which causes an electrical short circuit and reduces the safety of the batteries, is a significant issue. The dendritic growth is an intrinsic property of the alkaline or alkaline earth metal ions; therefore, it is very difficult to solve as long as these metal ions are used as a charge carrier. On the other hand, the use of molecular ions does not cause this problem in principle. These characteristics will dramatically improve the safety of high voltage batteries.

### Minor Metal Free

Elemental lithium is indispensable as the carrier ion in rechargeable lithium batteries; however, it is also a minor metal element. Using molecular ions consisting of more abundant elements can be an option for developing a minor metal-free battery system.

To enjoy the above mentioned benefits of molecular ion batteries, the development in the active materials of both the positive- and negative-electrodes is indispensable. Searching for a negative electrode active material exhibiting a low redox potential is especially significant. In this paper, we chose organic compounds for both electrodes; however, the properties of storing molecular ion charge carriers are not limited to organic compounds, so that conventional inorganic materials can also be a candidate. We believe that further material research will pave the way for the progress of this new battery system.

## Methods

### Materials

All the reagents were commercially obtained and used without further purification. The suppliers of the major reagents are as follow: tetrabutylammonium hexafluorophosphate (*n*-Bu_4_NPF_6_) (Tokyo Kasei), propylene carbonate (Kishida Chemical), poly(*N*-vinylcarbazole) (PVK) (average molecular weight: 1.1 × 10^6 ^g mol^−1^) (the Sigma-Aldrich Corp.), 4,4’-bipyridine (Tokyo Kasei), 1,5-dibromopentane (Tokyo Kasei), and ammonium hexafluorophosphate (Wako).

### Synthesis of the negative electrode polymer (poly(1,1’-pentyl-4,4’-bipyridinium dihexafluorophosphate: PBPy)

1,5-Dibromopentane (0.4 mL, 3.0 mmol) was added to an *N,N*-dimethylformamide solution (3 mL) of 4,4’-bipyridine (0.47 g, 3.0 mmol). The solution was stirred for 2 h at 150 ^o^C. The resulting precipitate was filtered and washed with ethyl acetate to give a crude solid of the bromide salt of the polymer (1.2 g, 94% yield). A portion of which (0.20 g) was dissolved in water (2.0 mL), and to which an aqueous solution (1.5 mL) of ammonium hexafluorophosphate (0.80 g, 4.8 mmol) was dropwise added at room temperature. The precipitate was collected by filtration and washed with water. The obtained material was recrystallized and dried under vacuum at 120 ^o^C for 1 h to yield PBPy (0.17 g, 62% yield). The prepared material was characterized by a melting point measurement (OptiMelt MPA-100, Stanford Research Systems, Inc.), NMR spectroscopy (JEOL, JNM-ECA series, *ν*(^1^H) = 500 MHz), and elemental analysis. M.p.: 300 ^o^C. ^1^H-NMR (DMSO-*d*_6_): *δ* 9.4 (4H, pyridinium-CH), 8.8 (4H, pyridinium-CH), 4.7 (4H, pentyl-CH_2_), 2.1 (4H, pentyl-CH_2_) 1.5 (2H, pentyl-CH_2_) (Supplementary Figure S5a). ^13^C-NMR (DMSO-*d*_6_): *δ* 149 (pyridinium), 146.2 (pyridinium), 127.1 (pyridinium), 61.1 (pentyl), 30.7 (pentyl) 22.9 (pentyl) (Supplementary Figure S5b). Anal. calcd for (C_15_H_18_F_12_N_2_P_2_)_n_: C, 34.90; H, 3.51; N, 5.43%. Found: C, 34.56; H, 3.69; N, 5.17%.

### Preparation of electrodes and cells

The electrodes and coin-type sealed cells for the battery tests were prepared as follows. A slurry for the positive electrode was first prepared by mixing PVK, acetylene black (denka black), vapor-grown carbon fiber as the conductive additives, and PVDF as the binder in the weight ratio of 5:2:2:1 with *N*-methylpyrrolidone. The prepared slurry was placed on a chemically etched aluminum-sheet current collector, and the resultant electrode was dried, then roll-pressed. The amount of the positive-electrode active material deposited was approximately 2 mg per electrode. A negative-electrode composite sheet was prepared by mixing the synthesized PBPy polymer powder, acetylene black as the conductive additive, and polytetrafluoroethylene as the binder in the weight ratio of 4:5:1. The sheet was then pressed onto an aluminum mesh current collector. The amount of the deposited negative-electrode active material was approximately 8 mg per electrode.

As for the half cell test, the prepared positive and negative electrodes were examined by making three-electrode type half-cells in a tetrabutylammonium hexafluorophosphate (*n*-Bu_4_NPF_6_) / propylene carbonate (PC) system (1 mol L^−1^) with an activated carbon counter electrode and the Ag^+^/Ag reference electrode consisting of an Ag wire in a glass tube carrying a porous Vycor tip filled with 0.01 mol L^−1^ AgNO_3_ / 0.1 mol L^−1^ tetraethylammonium perchlorate in acetonitrile. The half-cells were prepared under a low humidity environment (dew point < −70 ^o^C).

A full-cell composed of the positive and negative electrodes was prepared as follows. First, the electrodes were pretreated by applying a given charge/discharge pattern before the full cell assembly. As for the positive electrode, a dozen charge/discharge cycles was applied and then the cycles stopped in the charged state. The negative electrode was charged only once. The pretreated positive-electrode and negative-electrode (both charged) were removed from the half-cells, and then placed in an IEC R2032 coin-type cell case with a glass filter separator. After the electrolyte solution was added, the cell case was sealed. The full-cell was prepared under an inert atmosphere.

### Charge/discharge test

For the charge/discharge cycle-life test of the half-cells, the prepared cells were galvanostatically charged and discharged at the current density of 100 mA g^−1^ in the potential range of +1.8-−0.2 V *vs.* Ag^+^/Ag, and −0.2-−1.8 V *vs.* Ag^+^/Ag, for the positive and negative electrodes, respectively, at 20 ^o^C. As for the full-cell test, the cell was also galvanostatically discharged and charged at the current density of 100 mA g_(PVK)_^−1^ in the potential difference (voltage) range of +3.0 - 0.0 V at 30 ^o^C. The high-rate capability of the full-cell was examined by discharging at various current densities after several cycles (Supplementary Figure S4). Furthermore, an intermittent high-rate performance was also evaluated by discharging for 5 s at various current densities in the fully charged state. All of the charge/discharge tests were performed using computer-controlled systems (BLS series, Keisokuki Center Co., Ltd.) (ABE system, Electrofield Co., Ltd.). In this paper, the obtained capacities are expressed in terms of per mass of the active materials.

### Elucidation of carrier ion

The variation in the charge carrier concentration of the electrode was quantitatively determined using an energy-dispersive X-ray spectrometer (EDX, JED-2300, JEOL) connected to a scanning electron microscope (SEM, JSM-6510LA, JEOL). As for the sample preparation, IEC R2032 coin cells (two-electrode type) were prepared with an activated carbon counter electrode. An electrode using PVK or PBPy as the active material was mixed with a given amount of silica or alumina powder as the internal atomic reference. The amount of the reference was adjusted to be equimolar to that of the nitrogen atoms in the active materials. The cells incorporating the prepared electrodes were also galvanostatically charged and discharged at the current density of 100 mA per gram of the active material in the voltage range of −0.3-+3.2 V and +0.4-+2.7 at 30 ^o^C for the positive and negative electrode tests, respectively. The electrodes removed from the cells after a given charge/discharge cycling were washed with degassed tetrahydrofuran to remove the residual electrolyte salt before the measurement. The relative intensity of the phosphorous atoms in the electrodes to the Al or Si signal from the internal reference was converted to the ratio of the nitrogen atoms.

### Theoretical calculations

A quantum chemistry calculation based on the density functional theory (DFT) was performed using the GAUSSIAN 03 program package to obtain a theoretical insight into the electronic states of the active materials. A combination of an unrestricted hybrid functional of UB3LYP and a basis set of 6-31G(d)^29^,^30^ was used for the geometry optimization of the cation radical state of *N*-methylcarbazole and the fully reduced 1,1’-dimethyl-4,4’-dipyridinium skeleton (1,1’-dimethyl-1*H*,1’*H*-[4,4’]bipyridinylidene) (Supplementary Figure S3b). For the energy comparison, a single point calculation was used for the optimized structures in which the self-consistent isodensity polarizable continuum model (SCI-PCM)^31^ under a high dielectric constant environment (*ε* = 47) was used to reduce the polar effect on their energy levels. The calculated MOs were visualized by Gauss View 3.0.

## Additional Information

**How to cite this article**: Yao, M. *et al.* Molecular ion battery: a rechargeable system without using any elemental ions as a charge carrier. *Sci. Rep.* **5**, 10962; doi: 10.1038/srep10962 (2015).

## Supplementary Material

Supplementary Information
