# Supplementary material for: Molecular ion battery: a rechargeable system without using any elemental ions as a charge carrier
Source: Sci Rep. 2015 Jun 4;5:10962. doi: 10.1038/srep10962 (PMC4455302; doi:10.1038/srep10962)
Supplement: Supplementary Information [file srep10962-s1.doc]

**Supplementary Information**

**Molecular ion battery: a rechargeable system**

**without using any elemental ions as a charge carrier**

Masaru Yao*, Hikaru Sano, Hisanori Ando, and Tetsu Kiyobayashi

Research Institute for Ubiquitous Energy Devices,

National Institute of Advanced Industrial Science and Technology (AIST)

1-8-31 Midorigaoka, Ikeda, Osaka 563-8577, Japan

* Corresponding author;

Tel: +81-72-751-9651; Fax: +81-72-751-9629; E-mail: m.yao@aist.go.jp

**Figure S1 | Typical charge/discharge curves of the cells with an activated carbon (AC) based counter electrode for the EDX measurement.** (a), The charge/discharge behavior of the PVK-AC cell. (b), The charge/discharge behavior of the AC-PBPy cell. The measured positions were marked with asterisks (*) and the notes of the redox state of each working electrodes.

**Figure S2 | EDX spectral change in the electrode during cycling.** (a), The change in the PVK-based positive electrode. (b), The change in the PBPy-based negative electrode. Each of the spectra was normalized by the intensity of the reference peaks (*: Al, **: Si).

**Figure S3 | Quantum chemical calculation.** (a), The structure of *N*-methylcarbazole as a model compound for the PVK positive electrode. (b), The structure of the fully reduced 1,1’-dimethyl-4,4’-dipyridinium skeleton as a model compound for the PBPy negative electrode. (c), Their molecular orbitals and the calculated energy diagram. Calculated at the UB3LYP/6-31G(d) level. The self-consistent isodensity polarizable continuum model under a high dielectric constant environment (*ε*=47) was used.

**Figure S4 | Rate capability of the prepared molecular ion battery.** (a) Discharge curves at various current densities. Inset numbers indicate the current density (A g^–1^). (b), Relationship between the discharge capacity and the current density. (c), Current-voltage (*I-V*) and current-power (*I-P*) plots.

**Figure S5. | NMR spectra of PBPy.** (a) ^1^H-NMR spectrum. (b), ^13^C-NMR spectrum. Marked peaks are ascribed to the residual hydrogen of the deuterated solvent (*) and contaminant water (**).
